# Dengue and Zika virus infection patterns vary among *Aedes aegypti* field populations from Belo Horizonte, a Brazilian endemic city

**DOI:** 10.1371/journal.pntd.0009839

**Published:** 2021-11-02

**Authors:** Raquel Soares Maia Godoy, Luiza dos Santos Felix, Alessandra da Silva Orfanó, Bárbara Aparecida Chaves, Paula Monalisa Nogueira, Breno dos Anjos Costa, Aline Silva Soares, Cinthia Catharina Azevedo Oliveira, Rafael Nacif-Pimenta, Breno Mello Silva, Ana Paula Duarte, Marcus Vinicius Guimarães de Lacerda, Wuelton Marcelo Monteiro, Nágila Francinete Costa Secundino, Paulo Filemon Paolucci Pimenta

**Affiliations:** 1 Instituto de Pesquisas René Rachou, FIOCRUZ, Belo Horizonte, Minas Gerais, Brazil; 2 Programa de Pós-Graduação em Biologia Celular, Universidade Federal de Minas Gerais, Belo Horizonte, Minas Gerais, Brazil; 3 Fundação de Medicina Tropical Dr. Heitor Vieira Dourado, Manaus, Amazonas, Brazil; 4 Programa de Pós-Graduação em Medicina Tropical, Universidade do Estado do Amazonas, Manaus, Amazonas, Brazil; 5 Programa de Pós-Graduação em Ciências da Saúde, FIOCRUZ, Belo Horizonte, Minas Gerais, Brazil; 6 Departamento de Ciências Biológicas, Universidade Federal de Ouro Preto, Ouro Preto, Minas Gerais, Brazil; 7 Instituto Leônidas e Maria Deane, FIOCRUZ, Manaus, Amazonas, Brazil; Beijing Children’s Hospital, Capital Medical University, CHINA

## Abstract

Dengue virus (DENV) and Zika virus (ZIKV) belong to the same viral family, the Flaviviridae. They cause recurring threats to the public health systems of tropical countries such as Brazil. The primary Brazilian vector of both viruses is the mosquito *Aedes aegypti*. After the mosquito ingests a blood meal from an infected person, the viruses infect and replicate in the midgut, disseminate to secondary tissues and reach the salivary gland (SG), where they are ready to be transmitted to a vertebrate host. It is thought that the intrinsic discrepancies among mosquitoes could affect their ability to deal with viral infections. This study confirms that the DENV and ZIKV infection patterns of nine *Ae*. *aegypti* field populations found in geographically separate health districts of an endemic Brazilian city vary. We analyzed the infection rate, disseminated infection, vector competence, and viral load through quantitative PCR. Mosquitoes were challenged using the membrane-feeding assay technique and were tested seven and fourteen days post-infection (early and late infection phases, respectively). The infection responses varied among the *Ae*. *aegypti* populations for both flaviviruses in the two infection phases. There was no similarity between DENV and ZIKV vector competencies or viral loads. According to the results of our study, the risk of viral transmission overtime after infection either increases or remains unaltered in ZIKV infected vectors. However, the risk may increase, decrease, or remain unaltered in DENV-infected vectors depending on the mosquito population. For both flaviviruses, the viral load persisted in the body even until the late infection phase. In contrast to DENV, the ZIKV accumulated in the SG over time in all the mosquito populations. These findings are novel and may help direct the development of control strategies to fight dengue and Zika outbreaks in endemic regions, and provide a warning about the importance of understanding mosquito responses to arboviral infections.

## Author summary

Dengue and Zika are neglected diseases caused by viruses transmitted to humans by mosquitoes (vector-borne diseases). The primary vector of both diseases is *Aedes aegypti*, a highly abundant mosquito in tropical countries and adapted to the urban habitat. The viral cycle in the vector starts when the mosquito bites an infected person and acquires the viruses through the blood meal. When the infected blood reaches the mosquito’s midgut, the viruses invade the epithelial cells and disseminate in several organs until they reach the salivary glands, enabling viral transmission to the next person. However, the mosquitoes have developed strategies to combat the viral invasion and dissemination in their body, making this journey a challenge to the viruses. Herein, we show that the mosquito responses against dengue and Zika viruses are distinct. In addition, mosquitoes from separate populations of the same city have different abilities to deal with the viruses in both cases, dengue and Zika infections. Our results show the diversity of responses that the mosquitoes may present to viral infections. These findings may better direct disease control strategies to combat dengue and Zika outbreaks in endemic regions.

## 1. Introduction

Dengue virus (DENV) and Zika virus (ZIKV) are single-stranded positive-sense RNA viruses that cause recurrent threats to public health systems in many countries located in tropical and subtropical zones of the globe. DENV is currently present in the Americas, Asia, Africa, the Caribbean and Pacific, with Brazil being considered the most affected country in 2020, followed by Paraguay, Mexico, Vietnam, and Malaysia in the top five rank of DENV cases [1]. Regarding to ZIKV, the regions at risk are the Americas, South-East Asia, and Western Pacific. As of July 2019, 87 countries and territories presented evidence of ZIKV transmission, including Brazil [2]. Both DENV and ZIKV belong to the *Flavivirus* genus, cycle between humans and mosquitoes of the genus *Aedes*, and are mainly spread through the bite of its principal vector, *Aedes aegypti* and, to a lesser extent, the secondary vector *Aedes albopictus* [3–6].

Dengue outbreaks are suggested to have firstly occurred in the French West Indies and Panama in the 1600s. After the 1960s, when DENV was introduced to the Americas, dengue cases strongly increased, and many epidemics arose [7]. Currently, DENV is the cause of the most important arthropod-borne viral infection worldwide, with about 100–400 million cases reported annually [8,9]. In the Americas, more than 3 million dengue cases were reported in 2019, followed by more than 2 million in 2020 [10]. In contrast with the long period encompassed from the first dengue outbreak until today, Zika presented its first outbreak less than two decades ago, in 2007, in Micronesia [11,12]. After that, new cases were only related in 2013 [13], when the virus reemerged, spread rapidly across the Americas, and subsequently caused a significant outbreak in Brazil in 2015. The global number of Zika cases reported annually is not well determined. However, it is known that in the Americas, there were more than 976 thousand cases of ZIKV infections in the period between 2016 and 2021 [14].

Both DENV and ZIKV have distinct circulating strains worldwide. The DENV complex is formed by four antigenically distinct virus serotypes (DENV-1, DENV-2, DENV-3, and DENV-4) [15], all of which circulate in Brazil [16]. Each of these serotypes may present multiple lineages [17], and it is known that these distinct serotypes co-circulate in some urban areas causing coinfections or subsequential cases involving more than one serotype, which increases the human risk of acquiring severe dengue symptoms [16,18,19]. Unlike DENV, ZIKV has had only two major lineages identified, an African and an Asian lineage, with the latter being the most widespread in Brazil [20]. All the strains of DENV and ZIKV can cause a wide spectrum of health disorders, such as severe flu-like symptoms, severe bleeding, organ impairment and or plasma leakage, nausea, aches, and pains (eye pain, typically behind the eyes, muscle, joint, or bone pain). The ZIKV has also been responsible for cases of microcephaly in newborns [21,22].

The extensive global distribution of *Ae*. *aegypti* [23] and the high vector competence of these mosquitoes that transmit DENV and ZIKV contribute to the broad range of regions at risk of dengue and Zika outbreaks. Multiple factors determine the development of the viral infection in the mosquito vector and the efficiency of the dissemination and subsequent viral transmission. Extrinsic factors, such as the room temperature; and intrinsic factors, such as the midgut microbiota [24–26] and the mosquito’s genetics, influence the virus-vector interactions [27–29]. The mosquito’s genetics phenotypically determines the action of their innate immune response against viral replication and spread [30], which implies that genetic variability may generate distinct abilities to deal with the viruses. These interactions are biologically complex and may affect the competence of different *Ae*. *aegypti* populations in transmitting specific virus strains or serotypes.

To establish an infection after the ingestion of the infected blood meal, the virus first must survive, infect and replicate in the midgut cells (midgut infection barrier, MIB) of the *Ae*. *aegypti* mosquito, then escape to the hemolymph (midgut escape barrier, MEB) to disseminate and reach other organs such as the salivary gland (SG). Once inside the SG, the virus can be transmitted to vertebrate hosts through the subsequent blood-feeding [29–32]. This interval of time of the virus life cycle inside the vector, from the ingestion of the infected blood meal until the vector becomes infectious, is called the “extrinsic incubation period (EIP)”. It is well known that the EIP for both viruses is strongly affected by extrinsic factors, such as fluctuations in room temperature [33,34]. At constant temperature conditions, EIP tends to reduce as the temperature increases [35,36]. However, concerning the effects of the intrinsic factors in the EIP, little is understood.

The mosquito’s geographic origin has been shown to influence the vector’s competence in transmitting viruses. The primary factors that may explain the variabilities in the viral susceptibility among the vector populations are their genetic differences and or intrinsic microbiomes [37], which may occur at macro- and micro-geographic scales [28,38–41]. Despite the impact of dengue and Zika diseases on the global health systems, only a few studies have evaluated the ability of *Ae*. *aegypti* to be infected and transmit DENV and ZIKV in the context of populational variability. Therefore, this study aimed to determine an assumed vector’s competence and other associated parameters, such as the infection rate, disseminated infection rate, and the viral load in distinct mosquito populations derived from nine well-defined *Ae*. *aegypti* field populations in Belo Horizonte, a large and long-standing endemic Brazilian city [42,43]. Mosquito eggs were collected in the field sites and the adult females derived from them at F3-F4 generations were used in experiments. We tested whether the infection responses of the populations change differentially depending on the post-infection period. As DENV and ZIKV belong to the same viral family [3], we investigated any association between them regarding the viral infection. As such, the determination of the patterns of DENV and ZIKV infection response and variability among mosquitoes may help global health systems understand how the intrinsic factors of these vectors drive their defense mechanisms against the *Flavivirus* invasion.

## 2. Methods

### Ethics statement

This study was conducted in accordance with the Manual for the Use of Animals, published by the Oswaldo Cruz Foundation, Ministry of Health of Brazil (Decree 3179). The Ethics Committee approved it for the Use of Animals, Oswaldo Cruz Foundation (approval number L-1715), and the Animal Research Ethics Committee at the Tropical Medicine Foundation Dr. Heitor Vieira Dourado (approval number 002380/2016).

### *Ae*. *aegypti* collection

The *Ae*. (*Stegomyia*) *aegypti* eggs were collected using ovitraps distributed in the health districts of Belo Horizonte, State of Minas Gerais, Brazil (latitude 19° 56’ S, longitude 43° 56’ W, and altitude of 915 m above sea level). The city is the capital of Minas Gerais, has 2,521,564 inhabitants [44], and is the sixth-largest city in Brazil and the third-most populous metropolitan area in the country. It is divided into nine health districts, named Northern, Northeastern, Eastern, Barreiro, South-Central, Western, Northwestern, Pampulha and Venda Nova [44,45]. A total of thirty ovitraps were positioned for 5 days in several points of the nine health districts considering previous knowledge of the presence of *Ae*. *aegypti*. The eggs from the thirty ovitraps (from 30 to 200 per ovitrap) derived from each district were mixed and allowed to hatch and then reared until the adult stage. The *Ae*. *aegypti* mosquitoes were selected, separated and each collection was named according to the health district of origin. The mosquitoes were kept at a controlled temperature of 28°C, 80% relative humidity, and 12 h/12 h light-dark photoperiod. They were raised until enough specimens (F3-F4 generations) were used for the experimental infections. All samples of the first-generation adults (parental generation) derived from the field-sampled eggs were checked for natural DENV and ZIKV infection status using Real-Time PCR (qPCR), as described below.

### Virus culture

The DENV-2 (GenBank accession number KP188569; https://www.ncbi.nlm.nih.gov/) and ZIKV (ZikaSPH2015) [46] viruses circulating in Brazil were used in the experiments. The two viruses were maintained and propagated in C6/36 cultures with Leibowitz-15 medium, supplemented with 2% inactivated fetal bovine serum, 20μg/mL gentamicin, 5 μg/mL amphotericin B, 200U/mL penicillin (Sigma Aldrich, St Louis) [47]. Before the vector experiments, the two multiplying viruses were quantified by virus titration on Vero cells [Plaque forming unit (PFU/ml)] using a standard plaque assay [48].

### DENV and ZIKV infections of *Ae*. *aegypti*

Before the infection experiments, representative sub-samples of the first generation of the *Ae*. *aegypti* adults were processed using qRT-PCR to confirm they were negative for natural viral infections. This examination of possible DENV or ZIKV natural infection of the mosquitoes was necessary since this study was developed with adult female mosquitoes derived from field-collected eggs from endemic areas. Approximately 500 five-day-old *Ae*. *aegypti* mosquitoes from each health district were divided into two groups and simultaneously infected with DENV or ZIKV via a membrane feeding assay (water-jacketed glass feeder device covered with Parafilm) with blood meals containing either of the viruses. Virus titers of 1 × 10^5^ plaque-forming units per mL from C6/36 cell culture supernatants of one of the viruses were mixed with fresh mouse blood (2:1) and offered to the mosquitoes as described elsewhere [48,49]. Mosquitoes were allowed to feed for 30 min on the infective blood meals. Fully engorged mosquitoes (at least 150 individuals from each health district) were separated and maintained with a 10% sugar meal *ad libitum* for 14 days. Two groups of 20 mosquitoes from each health district were checked for infections at 7 days post-infection (dpi) and 14 dpi. The period of 7 dpi and 14 dpi were named “early infection” and “late infection”, respectively, since the extrinsic incubation period (EIP) of DENV and ZIKV in *Ae*. *aegypti* are around 14 dpi at room temperature (around 28° C) [50–52].

### Extraction and quantification of viral RNA by real-time PCR

Ten mosquitoes that had been experimentally infected with DENV or ZIKV from each health district were randomly chosen at 7 dpi and 14 dpi. These were anesthetized on ice and dissected under a stereoscope. Their bodies and head/SG (head with the attached salivary gland) were individualized and transferred to separate microtubes. According to the manufacturer’s instructions, the viral RNA was extracted from each sample using the QIAamp viral RNA mini kit (Qiagen) and subsequently stored at -70°C. The detection of viral copies was performed using qRT-PCR. The reaction was conducted in a 7500 Fast Real-Time PCR machine (Applied Biosystems) using the TaqMan Fast Virus 1-Step (Thermo Fisher Scientific). The viral RNA extracted from the bodies and head/SG of female mosquitoes were tested. All the analyses were performed in duplicate with standard curves, and with positive and negative controls. The negative controls were mosquitoes from the health districts that had been submitted to a non-infective blood meal, and the positive ones were *Ae*. *aegypti* mosquitoes from a colonized PP strain used routinely and always presents viral detection after an infective blood meal [26].

### Infection rate, disseminated infection rate and vector competence

The infection rate (IR), disseminated infection rate (DIR), and vector competence (VC) were determined for DENV and ZIKV to characterize the infection pattern of the nine mosquito populations of Belo Horizonte. The IR was calculated as the proportion of infected mosquito bodies related to the total number of tested mosquitoes. The DIR was the proportion of infected mosquito heads/salivary glands (head/SG) related to the number of infected mosquito bodies. The VC was the proportion of infected mosquitoes with viral detection in the head/SG to all tested mosquitoes [28]. Mosquitoes were not assayed for salivary gland infection or actual virus transmission. Therefore, the VC was assumed to be the same as the rate of virus dissemination to the head/SG tissues.

### Statistical analysis

Two-way ANOVA (multiple comparisons) and Tukey’s range test were used to compare the IR, DIR, and VC values among the early and late DENV and ZIKV infections (7 dpi and 14 dpi) considering all possible combinations. Mann-Whitney U tests were used to evaluate significance among viral load medians in the bodies and head/SG of each DENV and ZIKV infected mosquito population at 7 and 14 dpi. Similarly, the data from each population were analyzed together as representative of the entire city mosquito population. The evaluation of the body viral loads and head/SG viral loads among all populations was performed using Kruskal-Wallis one-way ANOVA tests. All statistical analyses were performed in GraphPad Prism, version 7.00 (La Jolla, CA, USA), and P values ≤ 0.05 were considered statistically significant.

## 3. Results

### Variation in the DENV infection of the *Ae*. *aegypti* populations

#### DENV early infection

**Fig 1:**
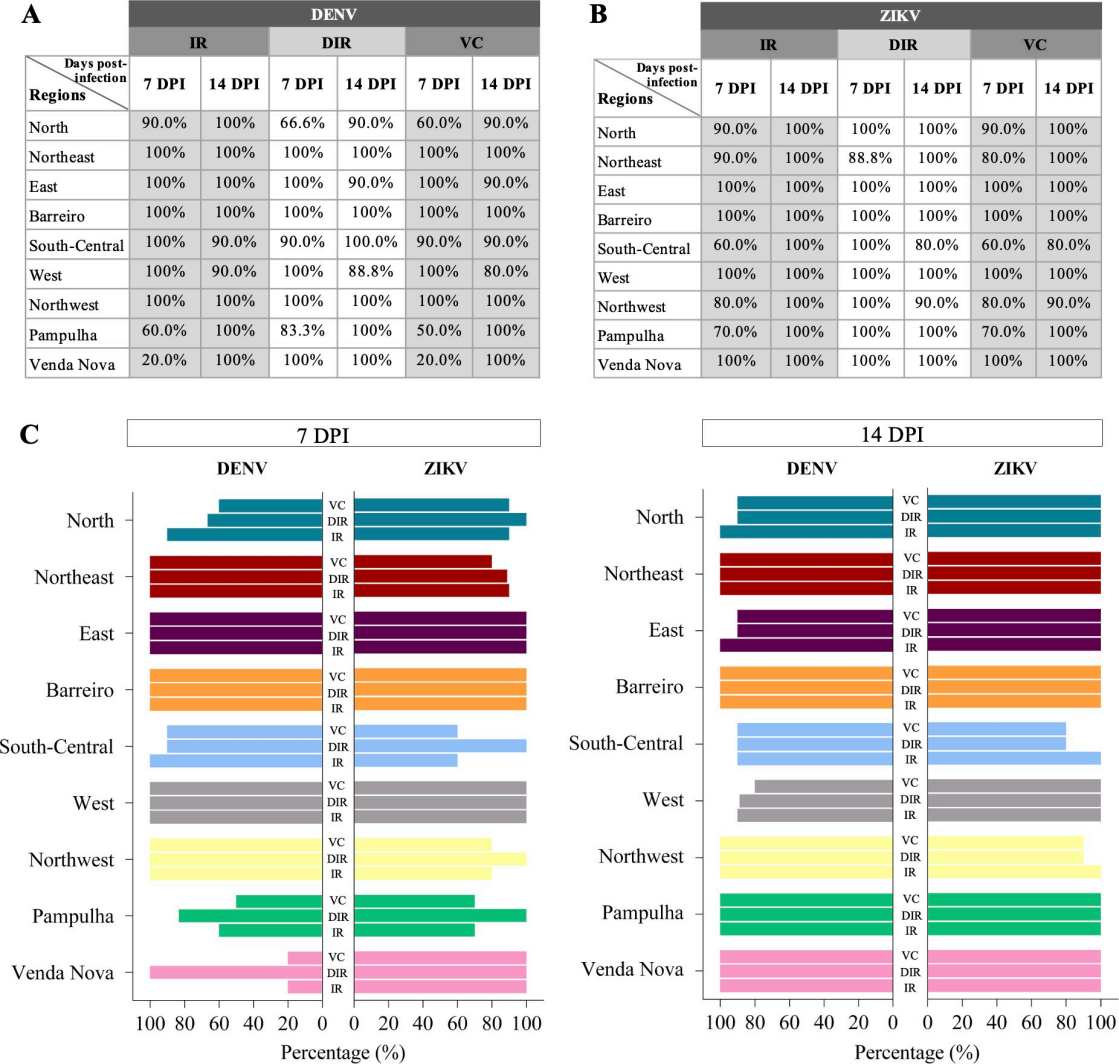
IR, DIR, and VC for the nine *Ae*. *aegypti* populations infected with DENV and ZIKV, at 7 and 14 days post-infection (dpi). **A-B**: Values of IR, DIR, and VC for the *Ae*. *aegypti* populations infected with DENV (**A**) and ZIKV (**B**). **C**: Comparison of the pattern of IR, DIR, and VC sets for each population, previously described in A-B, between DENV and ZIKV at 7 and 14 dpi. The bars represent the rates and were disposed of in a model that highlights each population’s specific patterns. No association is detected between DENV and ZIKV infection patterns of the populations, neither in 7 nor in 14 dpi. IR: Infection rate, the proportion of infected mosquitoes of the total number of mosquitos tested. DIR: Disseminated infection rate, the proportion of infected head/salivary gland (SG) of the total number of infected mosquitos. VC: vector competence, the proportion of infected head/SG of the total number of tested mosquitoes.

At 7 dpi, the DENV IRs for the mosquitoes from the Northeastern, Eastern, Barreiro, South-Central, Western, and Northwestern districts were 100%, and from the Pampulha and Venda Nova districts were 60% and 20%, respectively. The DIRs for mosquitoes from the Northeastern, Eastern, Barreiro, Western, and Venda Nova districts were 100%, and for the Pampulha, South-Central, and Northern districts, they were 83.3%, 90%, and 66.6%, respectively. The VCs for the mosquitoes from the Northeastern, Eastern, Barreiro, Western, and Northwestern districts were 100%, and for the Northern, Pampulha, and Venda Nova districts, they were 60%, 50%, and 20%, respectively (Fig 1A).

#### DENV late infection

At 14 dpi, the IRs became 90% for the mosquitoes from the South-Central and Western populations and the rest became 100%. The DIRs for the mosquitoes from the Northeastern, Barreiro, Northwestern, Pampulha, and Venda Nova districts were 100%; for the Northern, Eastern, and South-Central districts, they were 90%; and for the Western district, the DIR was 80%. The VCs for the mosquitoes from the Northeastern, Barreiro, Northwestern, Pampulha, and Venda Nova districts were 100% and, for the Northern, Eastern and South-Central districts, the VCs were 90%, while the VC for the Western district was 80% (Fig 1A).

### Variation in the ZIKV infection of the *Ae*. *aegypti* populations

#### ZIKV early infection

At 7 dpi, the ZIKV IRs for the mosquitoes from Eastern, Western, Barreiro and Venda Nova districts were 100%, from the Northern and Northeastern districts, these were 90%, and from the Northwestern, Pampulha, and South-Central districts, IRs were 80%, 70% and 60%, respectively. The DIRs were 100% among all mosquito populations except for those from the Northeastern district, which presented 88.8%. The VCs for the Eastern, Barreiro, Western and Venda Nova districts were 100%, for the Northwestern and Northeastern districts, these were 80%, and for the South-Central district, VC was 60% (Fig 1B).

#### ZIKV late infection

At 14 dpi, the IRs became 100% for all mosquito populations. The DIRs were 100% for all populations, except for those in the Northwestern and South-Central districts, with 90% and 80%, respectively. The VCs were 80% and 90% for the South-Central and Northwestern districts, respectively, and 100% for all other populations (Fig 1B).

**Fig 2:**
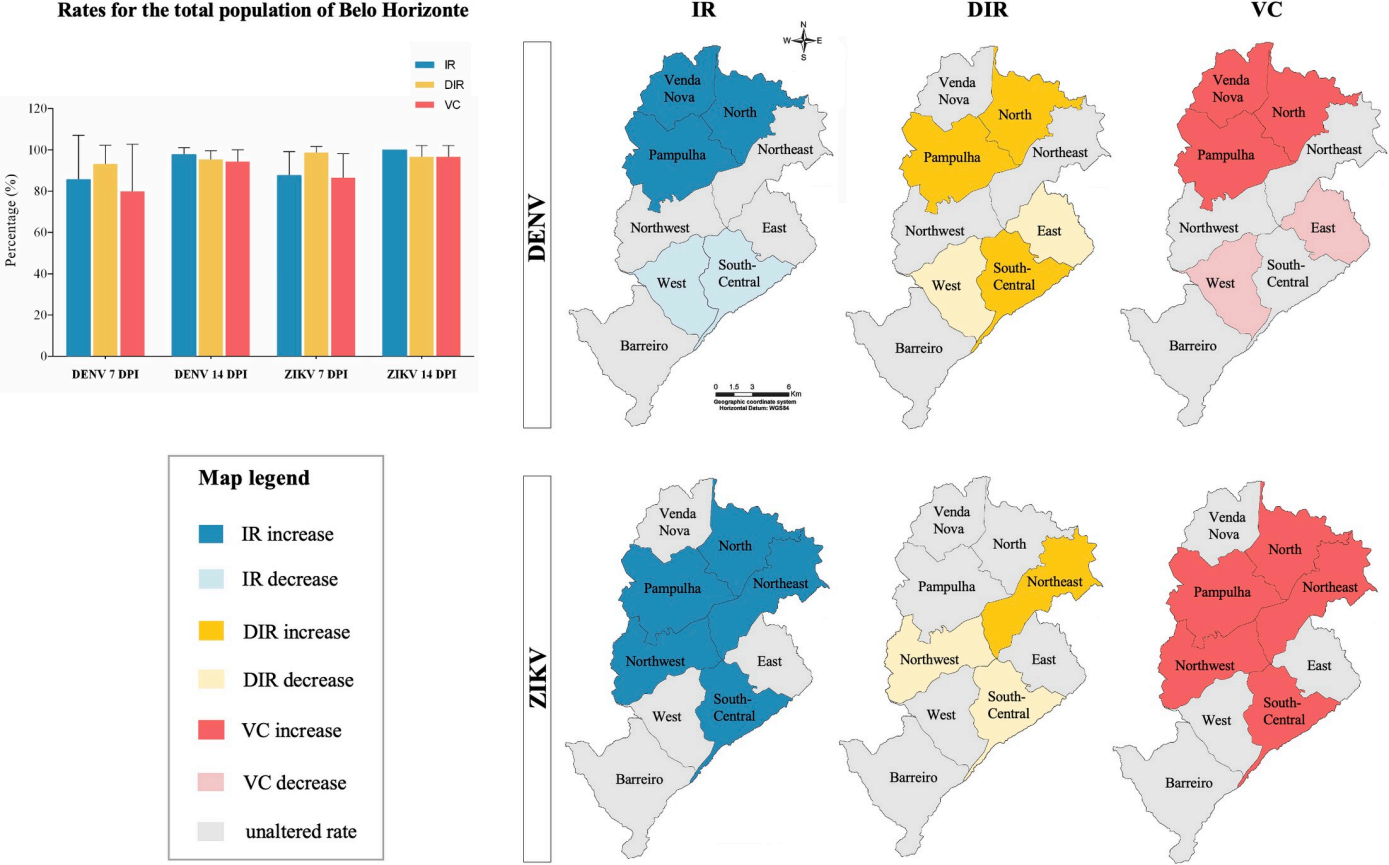
The graph shows the mean values of the infection rate (IR), disseminated infection rate (DIR), and vector competence (VC) for DENV and ZIKV at early infection (7 days post-infection—dpi), and late infection (14 dpi), considering the *Aedes aegypti* mosquitoes from all regions of Belo Horizonte. The schematic maps show the rates that increased, decreased or remained unaltered from 7 to 14 dpi. (The schematic maps were adapted from a previously published map [28], source: https://parasitesandvectors.biomedcentral.com/articles/10.1186/1756-3305-7-320/figures/2).

The IR, DIR, and VC rates of early and late infections of DENV and ZIKV for each *Ae*. *aegypti* population were statically analyzed and showed no equivalence among data collected from the different populations in both early and late infections (Fig 1C). The analyzed rates that increased, decreased or remained unaltered from 7 to 14 dpi are represented for each population in a map of the city of Belo Horizonte (Fig 2). In addition, considering the above rates for the total *Ae*. *aegypti* populations of the city (i.e., analyzing all health districts together), there were no significant differences among DENV and ZIKV infections in early and late infections.

### Viral RNA quantification in the *Ae*. *aegypti* populations

#### DENV early infection

At 7 dpi, the overall median of DENV RNA copies in the mosquito bodies from all nine populations measured using qPCR was 3 x 10^6^. The highest quantity of RNA copies was from the populations from the Barreiro and Western (2 x 10^8^) districts, and the lowest was from the populations from the Venda Nova district (zero). The other values (in ascending order) were from the Pampulha (2 x 10^2^), Northern (1 x 10^4^), South-Central (2 x 10^7^), Northwestern (3 x 10^7^), Northeastern (4 x 10^7^), and Eastern (1 x 10^8^) districts. In the nine populations, the overall median DENV RNA copies in all mosquito head/SG was 3 x 10^4^. The highest quantity of RNA copies was from the populations from the Northern district (2 x 10^8^), and the lowest one was from the populations from the Venda Nova district (zero). The other values (in ascending order) were from the South-Central (9 x 10^2^); Western (4 x 10^4^); Northwestern and Barreiro (1 x 10^5^); Eastern (7 x 10^5^); and Northeastern and Pampulha (1 x 10^6^) districts (S1A Fig).

**Fig 3:**
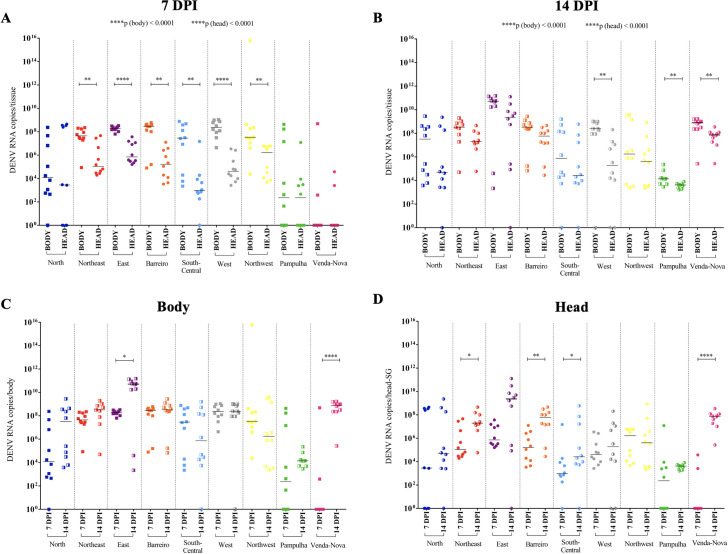
DENV viral load per body and head/salivary gland (SG) of *Ae*. *aegypti* at 7 and 14 days post-infection (dpi). **A**: DENV viral load per head/SG and body at 7 dpi in *A*. *aegypti* females from each district of Belo Horizonte. Each dot represents a tested female. **B**: DENV viral load per head/SG and body at 14 dpi in *A*. *aegypti* females from each district of Belo Horizonte. Each dot represents a tested female. **C-D:** Representation of the previous data **(A-B)** in another layout model to highlight the differences in DENV viral load between 7 and 14 dpi in the body (**C**) and head/SG (**D**) of the *Ae*. *aegypti* mosquitoes from each of the nine populations of Belo Horizonte. Fully filled and half-filled squares represent viral load in the mosquito bodies at 7 and 14 dpi, respectively. Fully filled and half-filled circles represent viral load in the mosquito head/SG at 7 and 14 dpi, respectively. P values > 0.05 (not significative) are not represented. P values ≤ 0.05, ≤ 0.01, ≤ 0.001, ≤ 0.0001 are summarized with one, two, three, and four asterisks, respectively.

The quantity of DENV RNA copies in the mosquito head/SG did not significantly differ from the bodies in the populations from the Northern, Pampulha, and Venda Nova districts (p > 0.9999, p = 0.5594, p > 0.9999, respectively). In contrast, the quantities of DENV RNA copies were greater in the mosquito bodies when compared to the head/SG for the Northeastern (**p = 0.0021), Eastern (****p < 0.0001), Barreiro (**p = 0.0021), South Central (**p = 0.0011), Western (****p < 0.0001), and Northwestern (**p = 0.0021) districts. The DENV viral load differ among the populations for both the body (****p < 0.0001) and head/SG (****p < 0.0001) (Fig 3A).

#### DENV late infection

At 14 dpi, the overall median of DENV RNA copies in the mosquito bodies was 2 x 10^8^ for all nine populations. The highest quantity of RNA copies was from the Eastern district (2 x 10^10^), and the lowest was from the Pampulha district (1 x 10^4^). In ascending order, the other values were from the South-Central (7 x 10^5^), Northwestern (1 x 10^6^), Northern (3 x 10^7^), Northeastern and Barreiro (3 x 10^8^), and Venda Nova districts (9 x 10^8^). The overall median DENV RNA copies in mosquito head/SG was 8 x 10^6^ for all nine populations. The greatest quantity of RNA copies was from the Eastern district (2 x 10^9^), and the lowest was from the Pampulha district (4 x 10^3^). The other values (in ascending order) were from the South-Central (2 x 10^4^), Northern (4 x 10^4^), Western (1 x 10^5^), Northwestern (4 x 10^5^), Northeastern (1 x 10^7^), Barreiro (6 x 10^7^), and Venda Nova districts (7 x 10^7^) (S1A Fig).

The quantity of DENV RNA copies in the mosquito head/SG did not differ significantly from the bodies in the populations from the Northern, Eastern, Barreiro, South-Central, and Northwestern districts (p = 0.2761, p = 0.0623, p = 0.0749, p = 0.4043, p = 0.3473, respectively). The DENV RNA copies were in greater quantities in the mosquito bodies than in the head/SG of the populations of the Western (**p = 0.0029), Pampulha (**p = 0.0078) and Venda Nova (**p = 0.0039) districts. The DENV viral loads differed among the populations for both the body (****p < 0.0001) and head/SG (****p < 0.0001) (Fig 3B).

The analyzed periods of the post-infection showed increases in the quantities of DENV RNA copies in the mosquito bodies from early to late infections in the populations of the Eastern (*p = 0.0232) and Venda Nova (****p < 0.0001) districts. No significant differences were detected for the Northern, Northeastern, Barreiro, South Central, Western, Northwestern, and Pampulha districts (p = 0.0627, p = 0.0786, p = 0.4727, p = 0.6706, p = 0.8928, p = 0.1639, p = 0.1720, respectively) (Fig 3C). In addition, there was an increase in the quantity of DENV RNA copies in the mosquito head/SG in the populations of the Northeastern (*p = 0.0172), Barreiro (**p = 0.0021), South Central (*p = 0.0238), and Venda Nova (****p < 0.0001) districts. No significant differences were detected for the Northern, Western, Northwestern, and Pampulha districts (p = 0.5252, p = 0.5149, p = 0.5191, p = 0., p = 0.0917, respectively) (Fig 3D).

#### ZIKV early infection

At 7 dpi, the overall median ZIKV RNA copies in mosquito bodies was 4 x 10^5^ for all nine populations when measured using qPCR. The greatest number of RNA copies was from the Northeastern district (4 x 10^6^), and the lowest was from the South-Central district (7 x 10^3^). The other quantities (in ascending order) were from Pampulha (1 x 10^5^), Barreiro and Western (2 x 10^5^), Eastern (3 x 10^5^), Northern (5 x 10^5^), and Venda Nova districts (7 x 10^5^). In all nine populations, the overall median ZIKV RNA copies for mosquito head/SG was 3 x 10^6^. The greatest quantity of RNA copies was from the Venda Nova district population (1 x 10^5^) and the lowest one was from the Pampulha district (7 x 10^1^). The other values (in ascending order) were from the Northeastern (9 x 10^1^), Barreiro and South-Central (1 x 10^2^), Northern (3 x 10^3^), Western and Northwestern (1 x 10^4^), and Eastern districts (3 x 10^4^) (S1B Fig).

**Fig 4:**
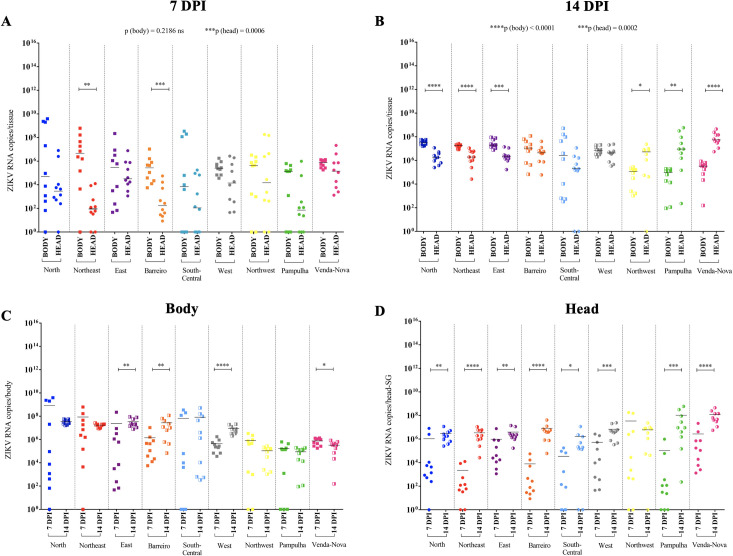
ZIKV viral load per body and head/salivary gland (SG) of *Ae*. *aegypti* at 7 and 14 days post-infection (dpi). **A**: ZIKV viral load per body and head/SG at 7 dpi in *Ae*. *aegypti* females from each district of Belo Horizonte. Each dot represents a tested female**. B**: ZIKV viral load per body and head/SG at 14 dpi in *A*. *aegypti* females from each district of Belo Horizonte. Each dot represents a tested female. **C-D:** Representation of the previous data **(A-B)** in another layout model to highlight the differences in ZIKV viral load between 7 and 14 dpi in the body (**C**) and head/SG (**D**) of the *Ae*. *aegypti* mosquitoes from each of the nine populations of Belo Horizonte. Fully filled and half-filled squares represent viral load in the mosquito bodies at 7 and 14 dpi, respectively. Fully filled and half-filled circles represent viral load in the mosquito head/SG at 7 and 14 dpi, respectively. P values > 0.05 (not significative) are not represented. P values ≤ 0.05, ≤ 0.01, ≤ 0.001, ≤ 0.0001 are summarized with one, two, three, and four asterisks, respectively.

The number of ZIKV RNA copies in the mosquito head/SG did not significantly differ from the bodies in the populations from the Northern, Eastern, South Central, Western, Northwestern, Pampulha, and Venda Nova districts (p = 0.3630, p = 0.9502, p = 0.4988, p = 0.1419, p = 0.8405, p = 0.2161, p = 0.1419, respectively). The ZIKV RNA copies were greater in number in the mosquito bodies than in the head/SG for the populations of the Northeastern (**p = 0.0021), and Barreiro (***p = 0.003) districts. The ZIKV viral load did not differ among the populations for the body (p = 0.2186), but differ for the head (***p = 0.0006) (Fig 4A).

#### ZIKV late infection

At 14 dpi, the overall median ZIKV RNA copies in mosquito bodies for all nine populations was 8 x 10^6^. The greatest quantity of RNA copies was from the Northern district (2 x 10^7^) and the lowest from the Pampulha district (9 x 10^4^). The other quantities (in ascending order) were from the Northwestern (1 x 10^5^), Venda Nova (3 x 10^5^), South-Central (2 x 10^6^), Western (7 x 10^6^), and Northeastern, Eastern, and Barreiro districts (1 x 10^7^). The overall median ZIKV RNA copies in mosquito head/SG for all nine populations was 4 x 10^6^. The greatest value of RNA copies was from the Venda Nova district (5 x 10^7^) and the lowest from the South-Central district (2 x 10^5^). The other quantities (in ascending order) were from the Northern and Northeastern (1 x 10^6^); Eastern (2 x 10^6^); Barreiro and Western (4 x 10^6^); Northwestern (5 x 10^6^); and Pampulha districts (8 x 10^6^) (S1B Fig).

The ZIKV RNA copies in the mosquito head/SG did not differ from the bodies of the populations of the Barreiro, South Central, and Western districts (p = 0.3104, p = 0.3056, p = 0.3104, respectively). In contrast, the quantities of ZIKV RNA copies were greater in the bodies than in the head/SG of the mosquitoes from the Northern (****p < 0.0001), Northeastern (****p < 0.0001), and Eastern districts (***p = 0.0002). However, the quantities of ZIKV RNA copies were smaller in the mosquito bodies than in the head/SG of the mosquitoes from the Northwestern (*p = 0.0354), Pampulha (**p = 0.0014), and Venda Nova districts (****p < 0.0001). The ZIKV viral load differed among the populations for both the body (****p < 0.0001) and head (***p = 0.0002) (Fig 4B).

The analyzed periods of the post-infection showed increases in the quantities of ZIKV RNA copies in the mosquito bodies from early to late infections in the populations of the Eastern (**p = 0.0021), Barreiro (**p = 0.0089), Western (****p < 0.0001) and Venda Nova (*p = 0.0431) districts. No significant differences were detected for the Northern, Northeastern, South Central, Northwestern, and Pampulha districts (p = 0.1883, p = 0.2761, p = 0.3030, p = 0.4237, p = 0.9115, respectively) (Fig 4C). In the mosquito head/SG, there was an increase in the number of ZIKV RNA copies in the populations from the Northern (**p = 0.0068), Northeastern (****p < 0.0001), Eastern (**p = 0.0021), Barreiro (****p < 0.0001), South Central (*p = 0.0125), Western (***p = 0.0007), Pampulha (***p = 0.001), and Venda Nova (****p < 0.0001) districts. No significant difference was detected for the Northwestern district (p = 0.2466) (Fig 4D).

**Fig 5:**
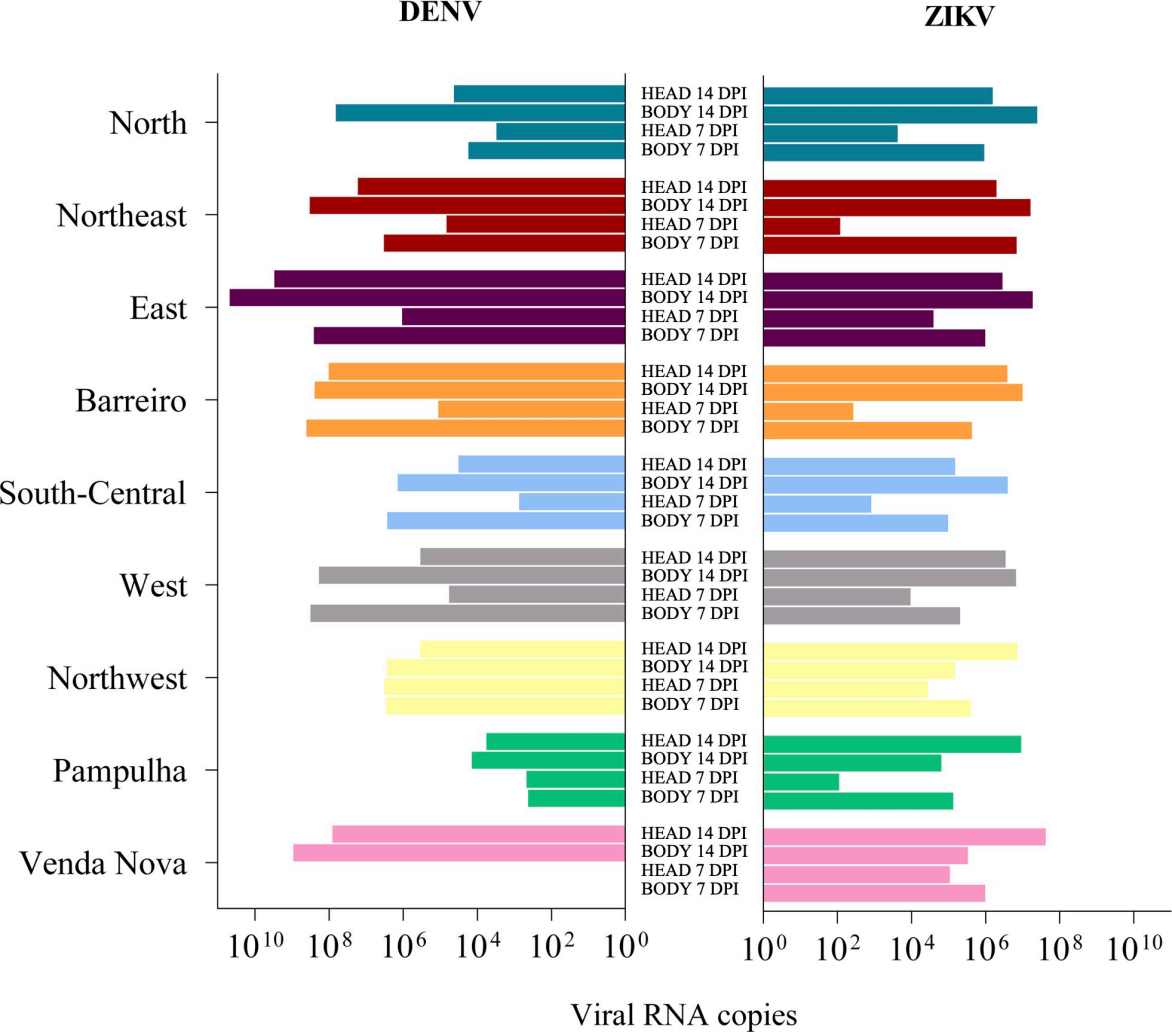
Comparison of the pattern of viral load sets for each population, previously described in Figs 3 and 4, between DENV and ZIKV in the body and head/salivary gland (SG) at 7 and 14 days post-infection (dpi). The bars represent the viral load medians, and were disposed in a model that highlight the specific patterns of each population.

**Fig 6:**
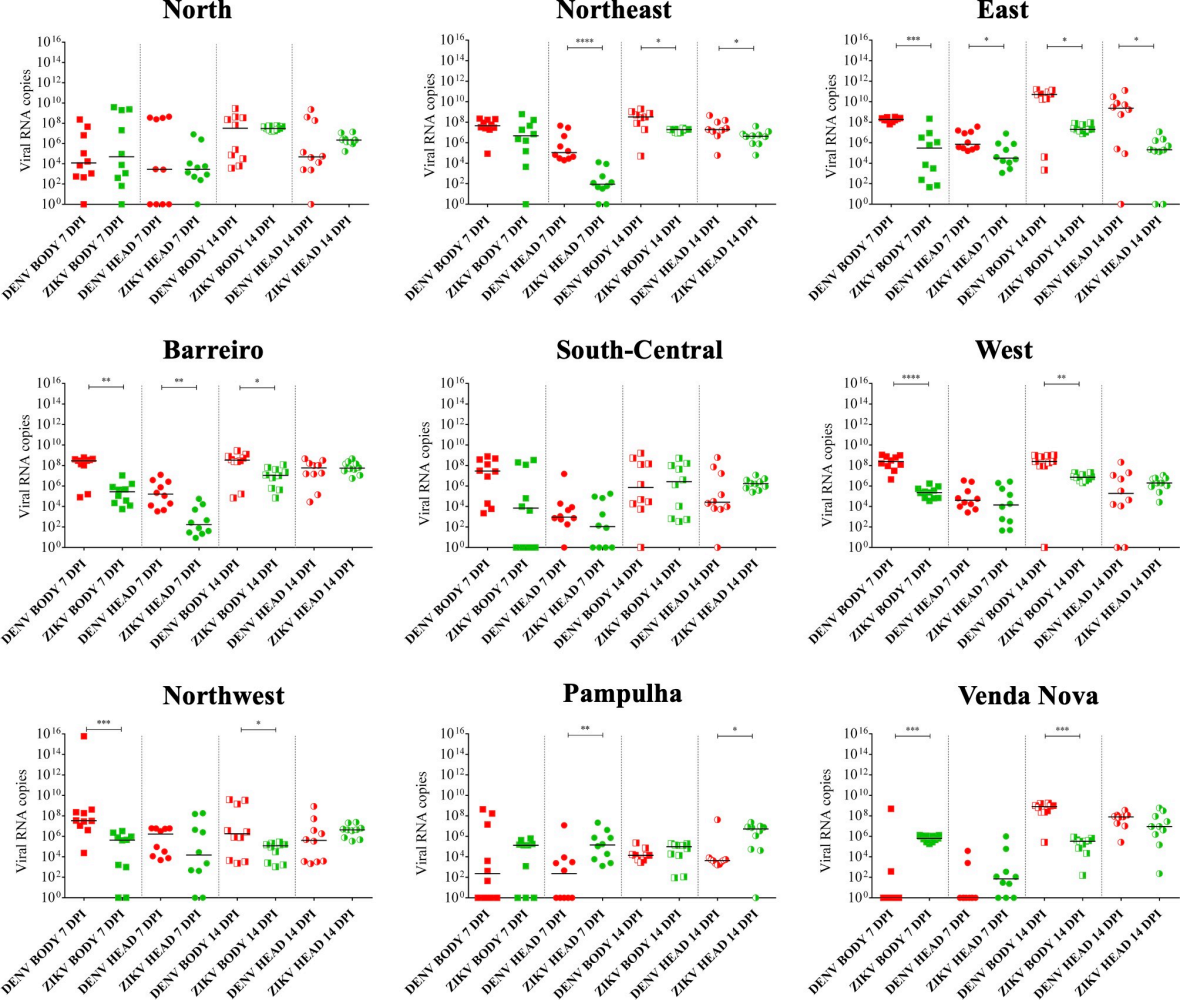
Representation of DENV (red) and ZIKV (green) viral load comparisons for each of the nine *Ae*. *aegypti* populations of Belo Horizonte. Fully filled and half-filled squares represent viral load in the mosquito bodies at 7 and 14 dpi, respectively. Fully filled and half-filled circles represent viral load in the mosquito head/SG at 7 and 14 dpi, respectively. P values > 0.05 (not significative) are not represented. P values ≤ 0.05, ≤ 0.01, ≤ 0.001, ≤ 0.0001 are summarized with one, two, three, and four asterisks, respectively. The p-values of the comparisons between body 7 dpi, head 7 dpi, body 14 dpi, head 14 dpi were, respectively: 0.7004, 0.9109, > 0.9999, 0.1419 (North); 0.0887, ****< 0.0.0001, *0.0133, *0.0349 (Northeast); ***0.0003, *0.115, *0.0232, *0.0286 (East); **0.0021, **0.0011, *0.0147, 0.7243 (Barreiro); 0.0703, 0.3484, 0.6706, 0.1419 (South-Central); ****< 0.0001, 0.3867, **0.0015, 0.4237 (West); ***0.0003, 0.2399, *0.0354, 0.1220 (Northwest); 0.7913, **0.0065, 0.3493, *0.0185 (Pampulha); 0.0008, 0.0734, ***0.0003, 0.2341 (Venda Nova).

In general, considering all the results, there was no equivalence between the DENV and ZIKV viral loads of the different *Ae*. *aegypti* populations of Belo Horizonte (Fig 5). Analyzing the viral loads between DENV and ZIKV infections for each population separately, all of them differ in at least two of the four comparisons (body 7 dpi, head/SG 7 dpi, body 14 dpi, head/SG 14 dpi). Except for Northern and South-Central populations, which did not vary in any comparison (Fig 6).

**Fig 7:**
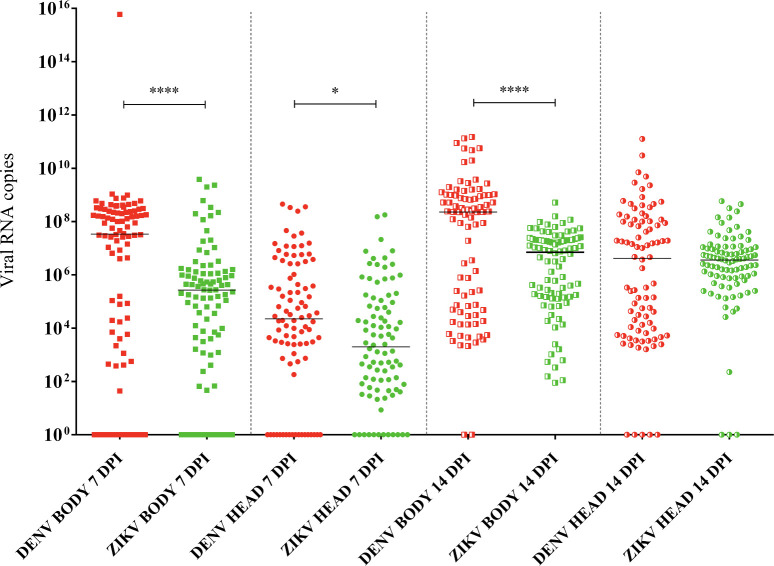
DENV (red) and ZIKV (green) viral loads for the total *Ae*. *aegypti* population of Belo Horizonte. The DENV total viral load in the body and head/salivary gland (SG) at 7 days post-infection (dpi) and in the body at 14 dpi is greater than the respective ZIKV total viral loads. At 14 dpi, the DENV total viral load in the head/SG is similar to that of ZIKV total viral load. Fully filled and half-filled squares represent viral load in the mosquito bodies at 7 and 14 dpi, respectively. Fully filled and half-filled circles represent viral load in the mosquito head/SG at 7 and 14 dpi, respectively. P values > 0.05 (not significative) are not represented. P values ≤ 0.05, ≤ 0.01, ≤ 0.001, ≤ 0.0001 are summarized with one, two, three, and four asterisks, respectively.

In relation to the viral load for the total population of Belo Horizonte, viral load for DENV was greater than that of ZIKV in the body for both 7 (****p < 0.0001) and 14 dpi (****p < 0.0001), and in the head/SG at 7 dpi (*p = 0.0117). No difference was detected between DENV and ZIKV viral loads in the head/SG at 14 dpi (p = 0.8726). The total DENV viral load was greater in the body than in the head/SG for both 7 (****p < 0.0001) and 14 dpi (***p = 0.0004). There was an increase in the total DENV viral load for both the body (**p = 0.0020) and head/SG (****p < 0.0001) from 7 to 14 dpi. The total ZIKV viral load was greater in the body than in the head/SG only at 7 dpi (****p < 0.0001), and no differences were detected at 14 dpi (p = 0.8177). There was an increase in the total DENV viral load for both the body (****p < 0.0001) and head/SG (****p < 0.0001) from 7 to 14 dpi (Fig 7). It was not identified mosquito groups from geographically closer districts with similar IR, DIR, VC, and viral loads for the two arboviruses. Finally, as expected, there was no viral load detection in the negative controls, and all the positive controls showed viral detection (S2 Fig).

## 4. Discussion

The susceptibility of *Ae*. *aegypti* populations from a long-standing Brazilian endemic city to DENV and ZIKV infection was evaluated at 7 and 14 days post-infection (dpi) in regards to the IR (infection rate), DIR (disseminated infection rate), and VC (vector competence). The IR indicates the virus’ ability to establish an initial infection in the vector midgut. The DIR is related to the virus’ ability to propagate and disseminate from the vector’s midgut to its secondary organs, in this case, the salivary gland (SG). In turn, the VC is directly proportional to IR and DIR (VC = IR x DIR) and indicates the proportion of mosquitoes in which the viruses crossed the SG barriers, thus completing their life cycle and becoming able to infect humans [28,38,53,54]. The results showed inter-population variations of the *Ae*. *aegypti* in the studied city considering the two arbovirus infections by DENV and ZIKV. All the populations had a relative quantity of individual mosquitoes that could develop infections and become competent at transmitting the two viruses at both early and late infection phases. The details and significance of these results are discussed for each arbovirus separately. The importance of the findings is described in a general context in the last topic.

### DENV infection susceptibility among *Aedes aegypti* populations

The analyses of the DENV infection responses showed that the Northeastern, Barreiro, and Northwestern health districts had the most competent dengue vectors among all the nine populations since the VC and the other analyzed parameters (IR and DIR) were 100% at both early and late infections. Differently, the VC increased from early to late infections in the Northern, Pampulha, and Venda Nova populations. These greater VC in the late infections were due to the increase in IR and DIR in the Northern and Pampulha populations. This means that the infection starts to be detected late in some mosquitoes (IR increase), and the viral dissemination to the heads occurs in more mosquitoes at the late infection phases. In contrast, in the Venda Nova district, the increase was only a reflection of IR increase (from 20 to 100%) during the post-infection period since the DIR was 100% in both phases of infection. This fact shows that the infection starts to be detected late in some mosquitoes. However, in both early and late infection phases, all infected mosquitoes were permissive to the viral dissemination to the head/SG. Finally, in the late infection, both Pampulha and Venda Nova districts’ populations reach a maximum VC of 100% and are highly susceptible to and competent at transmitting DENV. In the South-Central population, the VC was the same (90%) in early and late infections, though the IR decreased and the DIR increased over time. It indicates that DENV reached the head/SG of more individuals in late infection, but some individuals managed to eliminate the infection, thus establishing a type of compensation that generated unaltered VC during the post-infection period. Surprisingly, the VC decreased from early to late infection in the Eastern and Western populations, an event that was unexpected considering that DENV proliferates within the infection period (EIP) in infected vectors. The declining VC is related to reduced DIRs in both populations and reduced IRs in the Western population. Considering that, in the Eastern and Western populations, the virus was detected in all vector head/SG in early infection (DIR of 100% in both populations), we can infer that the declining DIR occurred because intrinsic barriers avoid viral spread and establishment of DENV in the late infection. The decrease in IR in the Western population indicates that some individuals were able to eliminate the virus. Probably, there was a rapid DENV dissemination that caused a sudden burst of the immune response of the vectors, thus negatively affecting the late infection. It is not possible to compare our data to make broader inferences from other ones. This study is the first to analyze the vector competence of distinct mosquito populations from the same endemic city. Therefore, further studies of different *Ae*. *aegypti* populations would be essential to investigate if this variability in vector competence also occurs in different mosquito strains.

Considering the *Ae*. *aegypti* populations’ susceptibility (permissiveness) to being infected by DENV and the viral dissemination in the mosquitoes’ tissues, it is possible to classify them in the following 4 infection patterns: **(a)** highly permissive with high dissemination at both early and late infections. **(b)** less permissive with high dissemination at the early phase, but highly permissive with high dissemination at the late phases; **(c)** less permissive with lower dissemination at the early infection phase, but highly permissive with high dissemination at the late infection phase; and finally, **(d)** highly permissive with high dissemination at the early infection phase, but less permissive with low dissemination at the late infection phase. These distinct types of response to DENV infection demonstrate that *Ae*. *aegypti* populations have wide-ranging patterns for fighting against these arboviral infections.

Considering the DENV loads in the infected mosquitoes’ tissues, the viral loads were similar or greater in the bodies compared to the head/SG. Thus, the number of DENV RNA copies in head/SG did not exceed the body’s respective values in any of the mosquito populations, either in early or late infections. Possibly, the viral load is more extensive in the body than in the head/SG due to the ability of DENV to replicate and disseminate in the hemolymph, reproductive system, muscle, and fat body, beyond the midgut, with the total virus accumulation in these tissues being high enough not to overcome the viral load in the head/SG. In addition, it was noted that the differences in the quantity of DENV in the mosquitoes’ bodies and head/SG in early infection decreased (Western) or ceased to exist (Northeastern, Eastern, Barreiro, South Central, and Northwestern) in late infections for most populations. In the Northeastern, Barreiro, and South-Central populations, the absence of differences between body and head/SG viral copy numbers in late infections could be attributed to the significant viral increase in the head/SG and no increase in the bodies. As Raquin and Lambrechts (2017) [55] have shown, DENV accumulated in the SG in *Ae*. *aegypti* overtime after infection, and these results were expected in some populations. For the other populations, where the viral load differences between body and head/SG were not detected (Northern) or increased (Pampulha and Venda Nova) from early to late infections. This fact may be attributed to the intrinsic differences among the populations that, in turn, determine different responses to DENV infection. The implication of these different infection responses is discussed in detail further on.

### ZIKV infection susceptibility among *Aedes aegypti* populations

The analyses of ZIKV infection responses showed that the Eastern, Barreiro, Western, and Venda Nova districts had the most competent vectors among the nine populations. The VC and the related IR and DIR rates were 100% in these mosquito populations in early and late infections. On the other hand, the VC increased from early to late infections in the Northern, Northeastern, South-Central, Northwestern, and Pampulha populations. This VC increase was mainly related to the increase in IR over time. These populations have an IR lower than 100% in early infections but equal to 100% in late ones, indicating that some mosquitoes need more than 7 dpi to develop the ZIKV infection completely. DIR also increased from early to late infections for the Northeastern population, indicating some of their mosquitoes are permissive to viral dissemination only in late infections. In contrast, the DIR decreased from early to late infections in South-Central and Northwestern populations, which indicates that some of their mosquitoes have a stronger blockade of viral establishment in the SG in late infections. Although the DIR decreased in South-Central and Northwestern populations, the increase in the IR overcame that and ensured the VC increase.

It is possible to classify 3 infection patterns considering the susceptibility of the *Ae*. *aegypti* populations to ZIKV and the viral dissemination in the mosquitoes’ tissues: (**a)** highly permissive with high dissemination at early and late infection phases; (**b)** less permissive with low dissemination at early infection, but high for both at late infection phases; and finally, (**c)** less permissive with low dissemination at early infection phases, but highly permissive with low dissemination at late infection phases. These different responses show that *Ae*. *aegypti* populations present a range of susceptibility patterns for fighting against ZIKV viral infections, although on a smaller scale than was seen for DENV.

For the viral load results, in early infections, the number of ZIKV RNA copies detected in the mosquito body was either similar or higher than in the head/SG when considering all populations. However, in late infections, three populations had higher viral levels in the head/SG than in the body, indicating that ZIKV reached the head/SG, replicated at high rates, and overcame the viral load in the body. From early to late infections, the amount of ZIKV increased in the body for four populations; however, it increased in the head/SG for all populations, except one. This fact demonstrates a strong tendency of ZIKV to colonize the SG during the infection process. Although the quantity of virus increased in the body over time after infection in some populations, indicating virus accumulation in the hemolymph and other body tissues, the increase in the head/SG was more representative and generalized, showing that the ZIKV accumulates markedly in the head/SG tissues over time.

### Differences between DENV and ZIKV infection patterns in *Ae*. *aegypti* and general conclusions

In the DENV infections, from early to late infections, the IR remained 100% in four populations, increased in three, and decreased in two of them. In contrast, for ZIKV infections, the IR was always 100% or increased in late infections. This fact indicates that ZIKV, once established in the body, remains after infection, but DENV can no longer be detected in the body over time in some infected mosquitoes. Therefore, ZIKV seemed to be more difficult to be eliminated from the mosquito body than DENV overtime after infection. In addition, for both DENV and ZIKV infections, some populations (which were not the same for the two arboviruses) had low IR at the early phase and the maximum IR (100%) at the late phase. This fact indicates the presence of a strong midgut barrier against viral establishment until 7 dpi that was not efficient for long periods, with the viruses managing to replicate later and be detected. Regarding the DIR, for both DENV and ZIKV, there was no trend towards a rate increase in late infections. Since the EIP requires 7–14 days for DENV and 7–10 days for ZIKV at the temperature in which the mosquitoes were incubated in the present study (28°C) [12,36,49], an increased DIR at 14 dpi was expected for both arboviruses, which did not occur. Therefore, DENV and ZIKV may not reach the SG in some mosquitoes or can reach them but are eliminated during the post-infection period. In the last case, it could be that the mosquito’s immune system managed to develop an efficient antiviral defense until 14 dpi, thus excluding the infection. Corroborating that cell events, such as apoptosis, phagocytosis, and autophagy, make significant contributions to the antiviral defense of dipteran insects like mosquitoes. This response occurs rapidly after infection, effectively reducing the duration of viral access to host factors that are crucial for replication [56].

Three DENV-challenged populations (33.3%) and five ZIKV-challenged populations (55.5%) were more competent at transmitting the viruses (higher VC) in late infections in detriment to early ones. Additionally, two populations were less competent in late infections than to early ones for DENV, while no decrease in competence was detected in ZIKV infections. This finding implies that the risk of ZIKV transmission from the mosquito to the vertebrate host tends to increase over time post-infection. However, the risk of DENV transmission can increase or decrease over time highly dependent on the assessed mosquito population. Interestingly, despite the strong differences seen for the infection responses among the populations, when the means of IR, DIR, and VC for the total population of the city are compared in sets, no difference is detected between DENV and ZIKV. This means that considering the city as a whole, it can be concluded that the competence of *Ae*. *aegypti* at transmitting DENV and ZIKV is similar, but through specific analysis of the populations, it is evident that the infections responses and VC values do not have equivalence between them. In a practical significance, the city is at the same risk to dengue and Zika outbreaks, but the human populations from each of the nine districts are subjected to different risks to acquire DENV or ZIKV. It demonstrates that the broader VC analysis may hide some important arboviral infection response characteristics noted in specific approaches.

In regards to the quantification of viral copies for both DENV and ZIKV, each tested population revealed that the viral load in the body remained high until 14 dpi, which indicates that despite the high viral spread to the head/SG, many viral particles remain in the other tissues of the body. Furthermore, the DENV viral load did not generally increase in the head/SG from early to late infections. Nevertheless, the ZIKV viral load of almost all mosquito populations (8 of the 9) increased in the head/SG. These results show that ZIKV strongly accumulates in SG tissue over time, but the DENV viral load does not have a pattern; and varies according to the different populations.

Considering the viral load comparisons between DENV and ZIKV infections for the total population of *Ae*. *aegpyti*, the higher DENV viral load in the body and head/SG in early infections and in the body in late infections related to the respective ZIKV loads is not reflected in VC values. As previously discussed, the analysis of the VC rates of the total populations of *Ae*. *aegpyti* revealed similar competencies for transmitting DENV or ZIKV. However, a higher viral load may confer an advantage to DENV through vertical transmission compared with ZIKV, since the viral permanence in high rates indicates larger viral dissemination to the secondary organs of the body, such as the ovary. Both arboviruses are able be transmitted to the offspring of *Ae*. *aegypti* through vertical transmission [57–59], however, as there are no studies comparing *Ae*. *aegypti* efficiency to vertically transmit DENV and ZIKV; therefore, no evidence was found to check that hypothesis.

Although there are some similarities in the general response patterns of DENV and ZIKV infections of the city mosquito populations when the susceptibility (permissivity) and the viral dissemination are considered; there was no equivalence between the two infections, neither in the analysis of the rate parameters nor in the analysis of the viral loads. Although two of the nine populations presented no difference between DENV and ZIKV loads, their IR, DIR, and VC rate sets varied. This fact shows that although DENV and ZIKV belong to the same viral family [3], the mosquito’s susceptibility to DENV and its respective mechanisms to control the infection is not similar to those of ZIKV. The variability in the IR, DIR, and VC rate sets among the populations was greater for DENV than for ZIKV. Similarly, the range of the viral loads was greater for DENV than for ZIKV, and the comparisons among the populations for the same tissue simultaneously showed a greater variability for DENV. It is known that virus-vector interactions include both, general and virus-specific components [60,61]. This means that some infection responses could be similar for DENV and ZIKV, but, as the vectors have evolved antiviral mechanisms in response to infection that are virus-specific, the susceptibility of *Ae*. *aegypti* has become substantially distinct between DENV and ZIKV infections. In this way, comparative transcriptomic analysis of the midgut between DENV- and ZIKV-infected *Ae*. *aegypti* at 7dpi showed 61% of the genes (135 of 223) to be uniquely regulated by ZIKV infection, whereas 35% (79 of 223) were regulated by both viruses in the same direction, and 4% in the opposite direction [62]. Besides, differently of DENV and other flaviviruses, which generally replicate in the cell cytoplasm, ZIKV antigen already was detected in the mosquito cell nuclei [63,64]. It indicates differences in the molecular interactions between the vector and each of the two viruses. Therefore, characteristics, such as the pathogen-associated molecular patterns, pathogenesis, or affected tissues, may vary depending on the specific arbovirus type [60], which could explain the differences seen for infection responses between DENV and ZIKV. Finally, the large range of different responses to DENV infection compared to ZIKV infection possibly points to the existence of a wider genetic divergence of the *Ae*. *aegypti* populations in regards to the phenotypic characteristics that are DENV-specific.

Belo Horizonte is one of the most forested capitals in Brazil, having around 600 thousand urban trees [65]. The city has an average annual temperature of 21.1°C and humidity of 72.2%, with altitudes ranging from 650 m in the Northeast to about 1,150 m in the South. The central area has the densest human occupation with the tallest buildings. The Southern, Southeastern, and Northeastern districts have the largest concentrations of green areas, which have a lower thermal load, favoring cooler temperatures at night. The positive and negative effects produced by the urban environment characteristics, such as geographical relief, temperature, sunlight, rain, wind, green areas, water bodies, streets, concentrations of buildings, etc., categorize the city as having eight classes of urban microclimate [66]. This condition demonstrates that the nine districts of Belo Horizonte, where the *Ae*. *aegypti* mosquitoes were collected in the present study, have distinct natural environmental conditions [67], which may be related to the inter-population differences in the vector’s infection responses that are phenotypically determined by variability in their physical barriers and/or their natural defense responses for confronting the arboviruses [68,69].

Differences in the environment influence population density, and the mosquito’s contact with vertebrate hosts, its nutrition, breeding water quality, predation, and intraspecific competition in the larval stage, which may direct the mosquito natural selection in different ways (different selective pressures), causing the variability [32,69]. Variation in population genetic parameters such as host costs of resistance, demographic population structure, pathogen virulence and transmissibility results in different types of selection, thus generating the pattern of genetic variation in vector susceptibility [70]. As a result, factors such as physical barriers efficiency and/or vector immunity vary and directly influence the vector’s ability to become infected after a infective blood meal, thus affecting the VC rate [26,32,68]. Corroborating the present results, previous studies have shown that susceptibility to arboviral infections varies in different *Ae*. *aegypti* populations [28,38,52]. Numerous studies of phylogeographic analysis have revealed that this vector have a high genetic diversity [28,38–41,71].

Another factor that may influence the inter-population differences in the vector’s infection responses is, to a lesser extent than genetic divergence, the presence of distinct microbial communities inside the insect’s midguts [37,60]. It is known that the *Ae*. *aegypti* microbiota may be vertically transmitted to their offspring and, in our study, the mosquito eggs were collected from distinct points of the city, which probably led to distinct microbial exposition to the parental generation of the mosquitoes. The use of further generations (F3/F4) reared in the laboratory conditions probably decreased the natural microbial divergence among the mosquito populations. However, as the microbiota of the mosquito’s midgut affects several mosquito traits, including development, nutrition, reproductive capacity, and VC [37,60,72], it is necessary to determine this possible interference in the infection response variability besides the genetic divergence.

Overall, our results demonstrated that all mosquito populations had individuals that were competent in transmitting DENV and ZIKV, which evidences a high potential for epidemic outbreaks in Belo Horizonte. There was considerable variability in the infection responses among the *Ae*. *aegypti* populations for both flaviviruses, which is probably a reflection of genetic differences between them. The VC increases or does not change in ZIKV infected mosquitoes over time after infection, but it increased, decreased, or remained unaltered in DENV infected mosquitoes. The viral load tended to persist in the body for both flaviviruses, even after a long post-infection period. The ZIKV showed a strong tendency to accumulate in the SG over the dpi in all populations, which did not occur for DENV. Finally, the infection patterns of the populations were not similar between DENV and ZIKV infections, indicating that, although both are flaviviruses, the mosquitoes orchestrate different defense mechanisms to fight them. The current lack of effective vaccines and treatments to dengue and Zika fever, and the mosquito resistance to population control strategies such as insecticides demonstrate the need for additional disease control strategies. The variability seen by our data points to the need to develop transmission-blocking strategies using broader approaches that work for a wide range of vectors. Since the understanding of the virus-vector interaction is critical for the epidemiological control and reduction of virus circulation, these findings should help to direct the development of control strategies to fight dengue and Zika outbreaks in endemic regions. Further studies could elucidate more details about the virus-vector interactions and the elements of these interactions that vary among genetically heterogeneous mosquito populations. A better understanding of the interrelationships of DENV and ZIKV with their vectors will provide a valuable perspective for developing effective approaches for controlling both viruses in the future.

## Supporting information

S1 Fig Number of DENV (A) and ZIKV (B) RNA copies (viral load) per body and head/salivary gland (SG) of *Ae*. *aegypti* at 7 and 14 days post-infection (dpi).
(TIF)

S2 Fig Negative and positive controls to validate the qPCR experiments of DENV (A) and ZIKV (B) infections. The RNA copies of each virus is represented in the body and head/SG, at 7 and 14 dpi, in *A*. *aegypti* mosquitoes from each administrative district and PP colonized strain (one individual of each) for the negative controls; and only from PP colonized strain for the positive controls (ten individuals).
(TIF)
