# Supplementary figures and images for: Dengue and Zika virus infection patterns vary among Aedes aegypti field populations from Belo Horizonte, a Brazilian endemic city
Source: PLoS Negl Trop Dis. 2021 Nov 2;15(11):e0009839. doi: 10.1371/journal.pntd.0009839 (PMC8562804; doi:10.1371/journal.pntd.0009839)

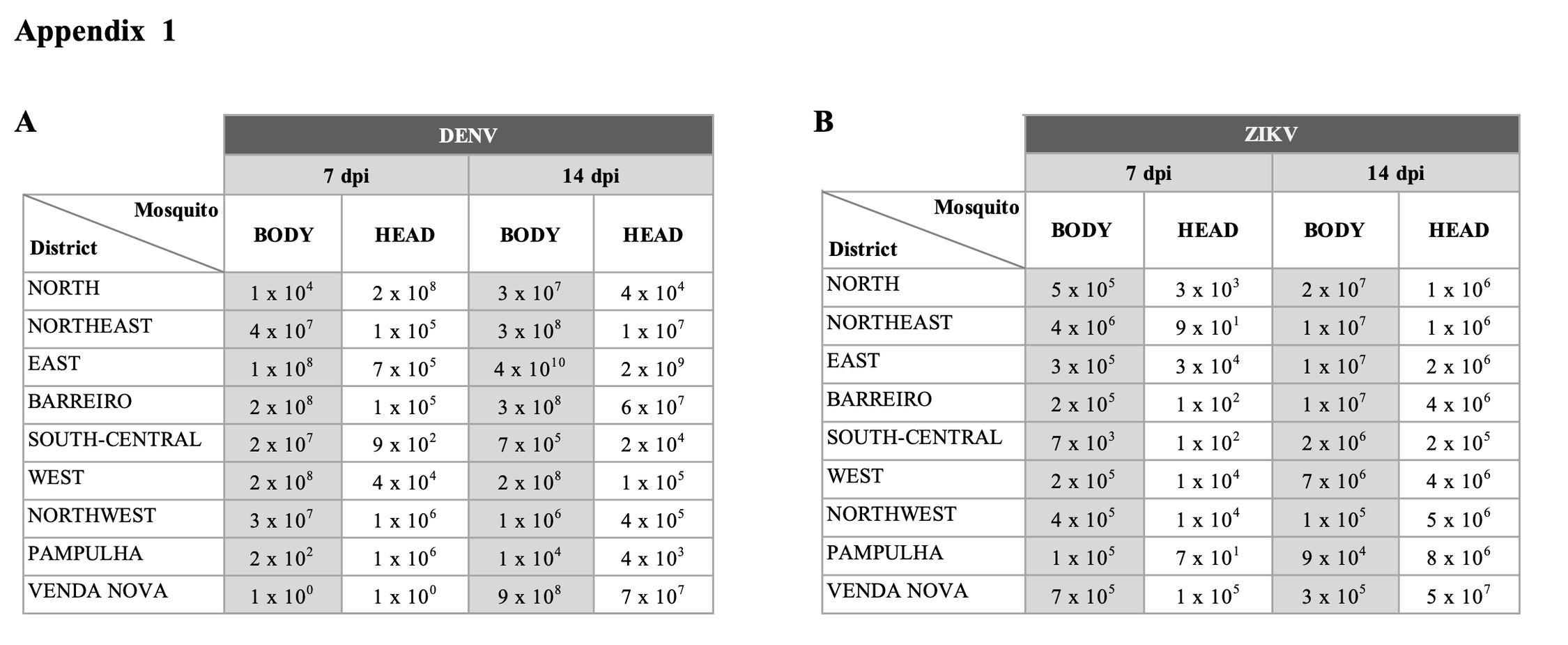

Supplement: S1 Fig — Number of DENV (A) and ZIKV (B) RNA copies (viral load) per body and head/salivary gland (SG) of Ae. aegypti at 7 and 14 days post-infection (dpi). (TIF) [file pntd.0009839.s001.tif]

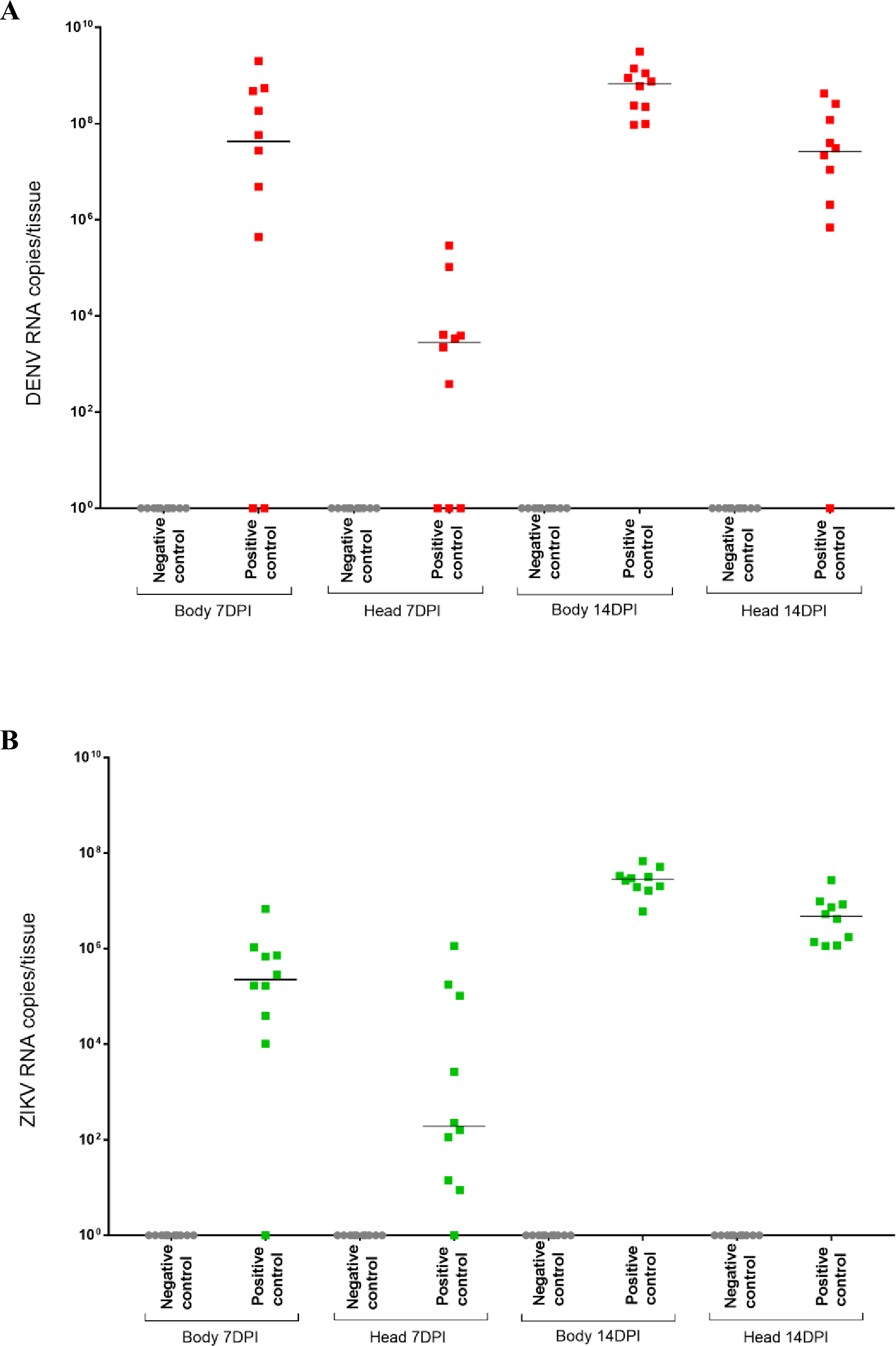

Supplement: S2 Fig — Negative and positive controls to validate the qPCR experiments of DENV (A) and ZIKV (B) infections. The RNA copies of each virus is represented in the body and head/SG, at 7 and 14 dpi, in A. aegypti mosquitoes from each administrative district and PP colonized strain (one individual of each) for the negative controls; and only from PP colonized strain for the positive controls (ten individuals). (TIF) [file pntd.0009839.s002.tif]
